# The Effects of Kindergarten and First Grade Schooling on Executive Function and Academic Skill Development: Evidence From a School Cutoff Design

**DOI:** 10.3389/fpsyg.2020.607973

**Published:** 2021-01-15

**Authors:** Matthew H. Kim, Sammy F. Ahmed, Frederick J. Morrison

**Affiliations:** ^1^Department of Psychology, University of Kentucky, Lexington, KY, United States; ^2^Institute for Social Research, University of Michigan, Ann Arbor, MI, United States; ^3^Department of Psychology, University of Michigan, Ann Arbor, MI, United States

**Keywords:** executive function, reading, math, school cutoff, quasi-experimental design, schooling

## Abstract

Early executive function (EF) skills reliably predict school readiness and future academic success. While children’s skills undergo rapid development during the transition to formal schooling, it remains unclear the extent to which schooling exerts a unique influence on the accelerated development of EF and academic skills during the early years of schooling. In the present study, a quasi-experimental technique known as the school cutoff design was used to examine whether same-aged children who made vs. missed the age cutoff for school entry significantly differed on EF, reading, and math outcomes. Data from 166 pre-k, kindergarten, and first grade children (Range = 3.75–7.58 years, 92 girls) from a longitudinal study of literacy development were analyzed. Children were assessed on EF, reading, and math skills in fall and spring. Results revealed unique effects of kindergarten, but not first grade, on growth in EF and reading over and above the effect of age. Schooling was unrelated to growth in math. Because kindergarten represents the first year of elementary school and children’s first exposure to a formal schooling environment, kindergarten schooling may be uniquely positioned to produce greater gains in academic and behavioral outcomes compared to other grades.

## Introduction

The transition to school is marked by dramatic changes at the individual and contextual levels. During the developmental period commonly known as the “5–7-year shift,” children across cultures develop increasingly sophisticated cognitive and social capacities (Sameroff and Haith, 1996). In addition to biological maturation, school-based intervention programs have been shown to improve domain-general cognitive skills such as executive functions (Weiland and Yoshikawa, 2013; Diamond et al., 2019) that are associated with academic outcomes (Ahmed et al., 2019). However, effective interventions often require substantial training and resources, and can be difficult to scale. As a result, increasing attention has focused on whether typical, practice-as-usual schooling can influence the development of executive function (EF) and academic skills.

While aspects of formal schooling—characterized by an emphasis on achievement, salience of peer comparisons, and greater expectations for independent work and self-regulated behavior—are frequently observed in first grade, recent trends indicate that these aspects emerge as early as kindergarten (Bassok et al., 2016). Therefore, examining whether kindergarten and first grade schooling exert unique influences on the development of children’s cognitive and academic abilities can yield insights into the complex interplay between context and development during the school transition period.

In Western societies where school enrollment is compulsory, disentangling the effects of schooling-related influences from non-schooling-related factors (such as age, parenting, and the home environment) was thought to be largely impossible. Because children cannot be randomly assigned to attend or not attend school, many studies have been mostly correlational in nature. However, the insight that many school districts enforce cutoff dates for kindergarten entry based on a child’s date of birth has led to the application of novel techniques such as the regression discontinuity design (RDD) and the school cutoff (SC) method (a form of RDD). These techniques take advantage of so-called natural experiments by dividing individuals into groups based on external factors, mimicking random assignment. By comparing outcomes between the two groups of children—who are virtually the same age but differ only in their schooling experiences—valid inferences regarding the causal impact of schooling on children’s outcomes can be drawn (Morrison et al., 2019).

Our understanding of the nature and magnitude of the causal impact of early schooling on academic and behavioral skills remains limited. While schooling has consistent effects on various aspects of literacy development, we know far less about the effects of schooling on math skills. Further, schooling effects on EF appear to depend on a number of factors, such as the nature of schooling, grade level, and how EF outcomes are measured. The present study seeks to add to this scholarly conversation by using a SC design to examine whether practice-as-usual kindergarten and first grade schooling has a causal impact on children’s EF, reading, and math skills, over and above factors unrelated to schooling.

### Schooling and EF Development

EF refers to a broad set of higher-order cognitive skills that enable individuals to exert control over basic cognitive processing skills to flexibly adapt to fluctuating environmental demands (Miyake et al., 2000; Posner and Rothbart, 2000). Although consisting of multiple distinguishable skills (e.g., working memory, inhibition, and cognitive flexibility), EF skills work in tandem to guide thoughts and behaviors by facilitating planning, problem solving, and enabling purposeful goal pursuits (Zelazo, 2015). This complex set of skills involves storing and manipulating information, inhibiting automatic responses to the environment, and focusing and shifting attention across multiple tasks, operations, or mental sets (Monsell, 1996; Miyake et al., 2000; Diamond, 2013). Research has identified multiple periods of accelerated growth in EF skills across the lifespan (e.g., Diamond, 2002; Zelazo et al., 2004; Jacques and Marcovitch, 2010; Montroy et al., 2016). However, the early periods of development have and continue to be a focal point of research given their relevance for school readiness and early academic outcomes. Specifically, EF skills that require the monitoring of overt, deliberate activities are particularly useful in a learning environment where students are constantly expected to monitor their behavior, pay attention, follow rules, and concentrate on various cognitive and behavioral tasks (Anderson, 2002; Blair, 2002; Blair and Razza, 2007; Diamond and Lee, 2011; Samuels et al., 2016).

Given the relevance of EF skills for supporting children’s learning and adaptation to school (e.g., Blair, 2002), there has been a growing interest in understanding the degree to which early schooling experiences contribute to children’s EF growth. Although the early schooling transition period has been characterized by rapid developmental changes in EF skills (e.g., Shing et al., 2010; Best et al., 2011; Wiebe et al., 2012; Willoughby et al., 2012), it remains unclear the degree to which schooling contributes to the observed acceleration of EF skills during this developmental stage, or is a result of influences unrelated to schooling. Estimating the role of schooling on children’s EF development can provide insight regarding the nature, timing, and development of these skills, and can inform instructional practices, curricula development, and targeted interventions. However, the few studies that have examined schooling effects on EF development using a school cutoff design have yielded mixed results. For example, Burrage et al. (2008) found pre-k and kindergarten effects on working memory development, but only a pre-k effect on inhibitory control. In contrast, Skibbe et al. (2011) found no effect of pre-k on behavioral self-regulation. In another study, Brod et al. (2017) found unique effects of first grade on EF task accuracy. In the present study, we contribute to this literature by exploring kindergarten and first grade schooling effects on children’s EF development using a global measure of EF.

### Schooling and Academic Development

Studies that investigated school- and age-related effects using RDD and the school cutoff method have focused largely on children’s academic skill development. The most studied academic domain across these studies has been various aspects of literacy development, from basic letter recognition and early phonological processing, to more advanced comprehension and writing skills (see Morrison et al., 2019, for a review). Not only have schooling effects been observed using intervention programs (Gormley et al., 2005; Weiland and Yoshikawa, 2013), but several studies have also demonstrated the effect of typical schooling programs on growth in reading and literacy skills across the transition to school (Morrison et al., 1995, 1997; Christian et al., 2000; Burrage et al., 2008; Skibbe et al., 2011; Kim and Morrison, 2018).

Much less is known, however, about the unique effects of typical schooling experiences on children’s mathematical development. Researchers have largely relied on highly specialized intervention programs to examine the impact of early schooling on children’s math skills. Using an RDD, Weiland and Yoshikawa (2013) found that participation in a prekindergarten program that used evidence-based curricula and a teacher coaching system led to statistically significant improvements in numeracy and math skills. In another study, Gormley et al. (2005) examined the causal impact of Oklahoma’s universal prekindergarten program using an RDD. The authors found that children who completed Oklahoma’s statewide prekindergarten program had significantly higher scores on standardized tests of math achievement than similar aged children that missed the cutoff for prekindergarten entry. While these studies suggest that early schooling can exert a unique effect on children’s math development over and above the effects of non-schooling-related factors, they are less informative for our understanding of the impact of typical, practice-as-usual schooling on children’s mathematical development.

Much of this work has focused on the impacts of pre-kindergarten given its role in preparing children for formal schooling (e.g., Wong et al., 2008). Fewer studies, however, have examined business-as-usual kindergarten and first grade schooling effects on children’s math development. One study using a SC design showed a causal impact of first grade schooling on math skills (Christian et al., 2000). However, because those children were assessed in the 1990s, it is important to acknowledge the significant policy changes that occurred as a result of the No Child Left Behind Act implemented in the United States in the 2000s. Due to the greater emphasis on academic standards, these accountability pressures are likely to have migrated to earlier grades, fundamentally altering the nature of early childhood education (Stipek, 2006). Indeed, recent research has revealed that instructional features typically encountered in first grade are now increasingly present in kindergarten classrooms (Bassok et al., 2016). The present study will provide insights into whether the initial years of formal schooling—kindergarten and first grade—have causal impacts on the development of academic and behavioral skills.

### Study Aims and Hypotheses

The present study builds on previous work examining the causal impact of schooling on literacy skills during the school transition period. Kim and Morrison (2018) examined the unique impact of pre-k, kindergarten, and first grade schooling on different dimensions of early literacy—decoding, phonological awareness, expressive vocabulary, and comprehension. While Kim and Morrison (2018) examined data from the same longitudinal study from which the present study is derived, the analytic method is significantly different between the two studies. Due to the relatively large sample size required for an adequately powered RDD, that study used a 6-month bandwidth and only examined end-of-year schooling effects based on group differences in literacy performance at a single time point. One significant limitation of the RDD was its inability to examine whether schooling effects extended not only to mean-level differences between the groups at the end of the school year, but also to growth in literacy skills throughout the year. In contrast, the present study used a SC design to examine both end-of-year schooling effects as well as differences in fall-to-spring growth, thereby extending this previous work to illuminate potential differences in growth trajectories in behavioral and academic skills during the school transition period.

In the current study, we set out to investigate whether typical kindergarten and first grade schooling has a causal impact on children’s EF and academic skills, over and above the effects of non-schooling-related factors. If a unique impact of schooling is observed, this would indicate that there might be some benefit—at least in the short-term—to enrolling children in kindergarten as soon as they are age-eligible for school entry. While various studies have consistently demonstrated positive impacts of early schooling on children’s literacy development, much less is known about the unique effects of typical schooling experiences on children’s mathematical development. However, given the intervention studies that demonstrate significant improvements in numeracy and math skills as a result of participation in prekindergarten programs, we hypothesized that kindergarten children would exhibit stronger end-of-year performance and stronger fall-to-spring growth in math skills compared to their same-aged pre-k peers. We expected to find a similar schooling effect for first grade children when compared to their same-aged kindergarten peers, and that these trends would also extend to literacy skills. For EF, despite the mixed findings in the literature based on EF subcomponents, we expected to see significant kindergarten and first grade schooling effects on a global measure of EF.

## Materials and Methods

### Sample

Data come from a longitudinal study of literacy development conducted in the mid-2000s. Children were recruited on a rolling basis from 13 elementary schools. Therefore, we examine data from pre-k, kindergarten, and first grade children assessed in the fall and spring over multiple years. The SC design compares two groups of children—“young” kindergarten (or first grade) children who made the cutoff for school entry, and “old” pre-k (or kindergarten) children who missed the cutoff—virtually identical in age but differing only in their schooling experiences. At the time of data collection, the age-cutoff enrollment date in Michigan was December 1. Previous studies have used either a 2-month (Burrage et al., 2008; Skibbe et al., 2011) or 3-month bandwidth (Brod et al., 2017; Zhang et al., 2018). In the present study, we adopted the more conservative 2-month bandwidth; the “made cutoff” group consisted of children born in October and November, while the “missed cutoff” group consisted of children born in December and January. However, we also conducted robustness checks to examine whether our results were sensitive to 1-month (more conservative) and 3-month (more liberal) bandwidths.

**FIGURE 1:**
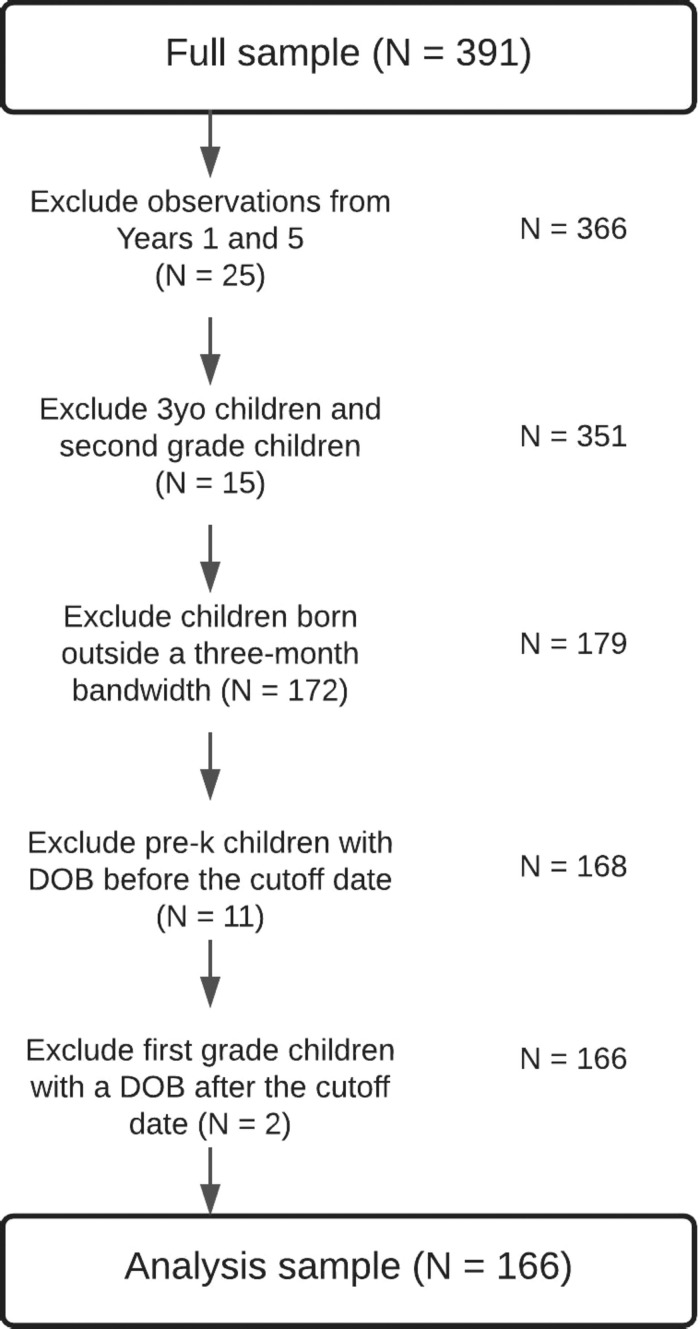
Flowchart of data and sample exclusions.

The complete data set consisted of 391 children. However, for purposes of the present analysis, not all children were eligible for study inclusion. Figure 1 presents a flowchart of data and sample exclusions. There were three exclusionary criteria: (1) Children who did not have data in the grade levels of interest, (2) Children born outside the cutoff window, and (3) Children in an unanalyzable group based on the child’s DOB relative to the cutoff date. Table 1 presents analytic sample sizes for each bandwidth. Due to the nature of the longitudinal study, children entered pre-k in years 2 and 3 of the study. Therefore, there were two separate cohorts of children examined in the present study. Data from both cohorts were combined prior to data analysis. Information on how the data were structured for data analysis can be found in the Supplementary Material.

**TABLE 1:** Sample sizes for each analytic group by bandwidth.

**Bandwidth**	**Total sample**		**Kindergarten effect**		**First grade effect**	
	**Missed cutoff**	**Made cutoff**	**Missed cutoff**	**Made cutoff**	**Missed cutoff**	**Made cutoff**
1 month	20	33	20	31	20	33
2 months	45	68	44	65	42	68
3 months	79	87	78	82	70	87

Children ranged in age from 3.75 to 7.58 years (*M* = 5.74 years); children’s ages at time of assessment at each wave are presented in Table 2. Demographic characteristics at baseline were as follows. Of the 166 children in the analytic sample, 74 were boys and 92 were girls. Of the 93 families who provided an open-ended response on race/ethnicity, 72 were White (77.4%) and 7 were African American (7.5%). Other responses were difficult to categorize due to the open-ended nature of the question. Of the 93 families who provided information on years of maternal education, 74 families (79.6%) reported at least 16 years of education, equivalent to a 4-year college degree. Of the 65 families who provided information on household income, the median income was $130,000 (Range: $40,000–$637,000).

**TABLE 2:** Average age in years at each data collection time point.

**Grade**	**Fall**	**Spring**	**Fall**	**Spring**	**Fall**	**Spring**
			
	**Year 2**		**Year 3**		**Year 4**	
Pre-kindergarten	4.62	5.19	4.84	5.39	N/A	N/A
Kindergarten	5.27	5.85	5.50	6.04	5.46	5.96
First grade	N/A	N/A	6.09	6.66	6.41	6.93

*Data reflect a 2-month bandwidth.*

*T*-tests were conducted to examine whether demographic characteristics differed between children eligible for the school cutoff study (i.e., born within 2 months on either side of the cutoff) compared to children who are not eligible. There were no significant differences between the two groups on child gender, *t*(164) = 0.77, *p* = 0.39; and household income, *t*(63) = 0.54, *p* = 0.27. There was a significant difference between the two groups on maternal education, *t*(91) = 2.70, *p* < 0.01, indicating that mothers of children with birth months in October through January (study eligible) had about 1 less year of education compared to mothers of children with birth months in February through September (study ineligible).

### Measures

#### Executive Function

EF was measured using a global, ecologically valid measure of self-regulation called the Head-Toes-Knees-Shoulders (HTKS; McClelland et al., 2014). In the HTKS, children respond behaviorally to a series of commands (“touch your head”) by doing the opposite of the stated command (child touches their toes). The task consists of two parts; Part I consists of a combination of two commands (e.g., “touch your head” and “touch your toes”) and Part II adds two additional commands (e.g., “touch your knees” and “touch your shoulders”) to increase task difficulty. In pre-kindergarten and kindergarten samples, HTKS performance is strongly correlated with each of the three dimensions of EF—cognitive flexibility, inhibitory control, and working memory—indicating strong construct validity with EF (McClelland et al., 2014). Children receive two points for a correct behavioral response and zero points for an incorrect response. If children initiate an incorrect movement but then self-corrects, the child receives one point. Parts I and II are each scored out of 20 points.

The HTKS was administered differently depending on the grade of the child. For pre-k children, only Part I of the HTKS was administered. For kindergarten and first grade children, Parts I and II were administered. This is in contrast to the current task guidelines which specify that children who score at least 4 points on Part I should proceed to Part II, regardless of the child’s age or grade (McClelland et al., 2018). This difference in task administration has potentially significant implications on scoring, which in turn has an impact on how schooling effects are calculated. Examining the effect of kindergarten schooling involves comparing pre-k children who missed the cutoff for school entry with kindergarten children who made the cutoff. Similarly, examining the effect of first grade schooling involves comparing kindergarten children who missed the cutoff for school entry (1 year prior) with first grade children who made the cutoff. Because kindergarten and first grade children both completed Parts I and II of the HTKS and have scores on the same metric, calculating the first grade effect is straightforward. However, the HTKS score for pre-k children is based only on Part I performance, while the HTKS score for kindergarten children is based on both Part I and II performance.

Two possibilities, imputation of missing data and variable standardization, are likely to generate biased estimates. First, Part II HTKS data could be imputed for pre-k children. While data that are missing by design can often satisfying the condition of data being missing completely at random, these situations typically deal with variations on matrix sampling, where different participants are purposely asked to complete surveys of different lengths (Graham et al., 2006). However, in this case, because all pre-k children did not complete Part II of the HTKS *by rule*, imputation of missing data is not appropriate. Second, converting HTKS scores to standard deviation units is another possibility. However, by standardizing scores, information on mean-level change over time is lost (Moeller, 2015). Because the school cutoff design involves assessing children’s growth from fall to spring, variable standardization is also not appropriate.

Instead, we pursue two alternative strategies in the present study. One strategy is to use only Part I HTKS scores for kindergarten children while disregarding their Part II scores. In this arrangement, both pre-k and kindergarten children would have HTKS scores on the same scale (out of 20 points). However, because most kindergarten children perform well on Part I, a ceiling effect might emerge for kindergarten children, which could artificially mask a kindergarten effect. Another strategy is to retain the HTKS scores as administered for pre-k (Part I only) and kindergarten children (Parts I and II). Because the upper bound for kindergarten HTKS scores is higher (40 points instead of 20 points), this strategy might exaggerate any observed kindergarten effect. However, this concern would be mitigated under the assumption that pre-k children, given their young age, would have been unlikely to perform well, or perhaps may not have even scored any points, on Part II. In the present study, we pursue the more conservative strategy of considering only Part I scores for both pre-k and kindergarten children. However, we also pursue the second strategy of retaining the HTKS scores as administered as a robustness check. In summary, two metrics of the HTKS were used: For pre-k children, a metric that only considers performance on Part I was used (HTKS20); for first grade children, a metric that considers performance on Parts I and II was used (HTKS40). For kindergarten children, both metrics were examined, with the HTKS20 being the preferred specification and the HTKS40 used as a robustness check.

#### Academic Skills

Literacy and numeracy skills were assessed using the Letter-Word Identification and Applied Problems subtests, respectively, of the Woodcock Johnson III Tests of Achievement (Woodcock et al., 2001). The Letter-Word Identification subtest (decoding) assessed word recognition, vocabulary, and reading skills by asking children to identify and pronounce letters and words with proper pronunciation. The questions became increasingly difficult as participant progressed through the task. The Applied Problems subtest assessed early math skills by presenting oral word problems pictures and numbers to children that assess basic mathematical concepts. Participants were asked to listen to each item, determine the procedure to solve the problem and successfully complete the computations. The task grew increasingly difficult and the participants were given a pencil and paper after they reached a certain point in the task to help solve the problems. External validation efforts have demonstrated excellent reliability (Cronbach’s α = 0.93) for the academic achievement battery among young students and very high test-retest reliability (*r* = 0.95; Woodcock et al., 2001). The WJ is specifically designed to generate scores that are comparable across ages, and empirical work has demonstrated that the basal and ceiling levels that determine the start and end rules are correctly defined (Watts et al., 2014). W scores were obtained using the WJ-III Compuscore software program. In contrast to metrics such as raw scores and standard scores, the W score metric is on an equal-interval scale and allows a direct comparison of achievement across different students, regardless of age (Jaffe, 2009). Means for the HTKS and WJ variables are presented in Table 3.

**TABLE 3:** Means and standard deviations for the HTKS and Woodcock Johnson academic measures.

**Variable**	**Pre-kindergarten**		**Kindergarten**		**First grade**	
	**Fall**	**Spring**	**Fall**	**Spring**	**Fall**	**Spring**
HTKS20	14.2 (7.5)	16.9 (5.4)	17.4 (4.2)	17.6 (4.8)	N/A	
HTKS40	N/A		28.7 (9.3)	32.1 (8.7)	34.6 (5.0)	36.5 (3.7)
WJ Letter-Word Identification W score	348.3 (17.4)	359.1 (24.8)	371.0 (28.1)	404.2 (28.6)	428.4 (34.3)	460.4 (25.6)
WJ Applied Problems W score	415.4 (10.8)	427.7 (15.2)	432.6 (15.9)	444.5 (15.8)	459.5 (16.7)	475.0 (18.4)

*Data reflect a 2-month bandwidth.*

### Data Analysis

We used a two-level nested linear mixed model, with time (fall and spring) as a within-subjects factor and group (e.g., pre-k and kindergarten; kindergarten and first grade) as a between-subjects factor. In addition to main effects of time and group, a Time × Group interaction would reveal whether there is an interactive effect of development and schooling (i.e., whether growth in EF or academic skills depend on whether the child missed or made the cutoff for school entry). Children were nested within classrooms that are nested within schools, and standard errors were clustered on schools and estimated using the delta method Dowd et al. (2014). Estimation was by maximum likelihood, and the model allowed for a random intercept and a random time slope for each child. Due to the small attrition rate, missing data were removed using listwise deletion. The xtmixed command in Stata 13 was used to analyze the data. Local effect size estimates (Cohen’s *f*^2^), appropriate for mixed effects linear regression models were generated using the method demonstrated in Selya et al. (2012) and reported for significant effects. Based on the guidelines described in Cohen (1988), *f*^2^***-***values of 0.02, 0.15, and 0.35 represent small, medium, and large effect sizes, respectively. The code used to generate the estimates can be found at https://osf.io/guh2f/.

### Balance Checks

Balance checks were conducted in order to determine whether the two schooling groups (i.e., made cutoff vs. missed cutoff) were equivalent on demographic characteristics such as gender, household income, and highest maternal education. When examining the two schooling groups that would determine the kindergarten schooling effect (i.e., pre-k children who missed the cutoff vs. kindergarten children who made the cutoff), there were no significant differences between the two groups on child age at testing, *t*(263) = 1.43, *p* = 0.15; child gender, *t*(265) = −0.40, *p* = 0.69; household income, *t*(98) = −0.72, *p* = 0.47; and years of maternal education, *t*(128) = −1.49, *p* = 0.14. When examining the two schooling groups that would determine the first grade schooling effect (i.e., kindergarten children who missed the cutoff vs. first grade children who made the cutoff), there were no significant differences between the two groups on child age at testing, *t*(302) = 1.11, *p* = 0.27; child gender, *t*(302) = −1.46, *p* = 0.15; household income, *t*(90) = 0.78, *p* = 0.44; and years of maternal education, *t*(119) = −1.50, *p* = 0.14. These results indicated that the two groups were essentially equivalent on demographic characteristics, strengthening our ability to make valid causal inferences regarding the unique impact of schooling on children’s academic and cognitive outcomes.

## Results

### Are There Kindergarten and First Grade Effects on EF?

**FIGURE 2:**
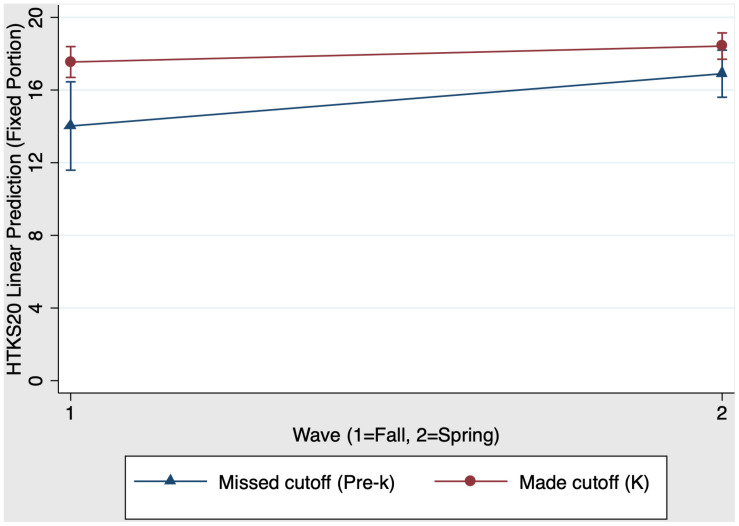
Kindergarten schooling effects on fall-to-spring change in HTKS20 scores.

We examined whether kindergarten schooling had a unique impact on children’s EF skills. We did this by performing a mixed-effects regression with group, time, and group × time predicting children’s HTKS20 scores. Results showed that the model was significant, Wald χ^2^(3) = 17.56, *p* < 0.001, indicating that the three-predictor model meaningfully predicted variability in HTKS20. Then, the contrast command—which permits ANOVA-style tests of main effects and interactions—was used. Figure 2 presents the kindergarten main effects and interaction on HTKS20 scores. There was a significant main effect of group, χ^2^(1) = 9.00, *p* < 0.01 (*f*^2^ = 0.08): Kindergarten children who made the cutoff for school entry have stronger HTKS20 scores than their same-aged pre-k peers who missed the cutoff. There was also a significant main effect of time, χ^2^(1) = 8.88, *p* < 0.001 (*f*^2^ = 0.10): Both pre-k and kindergarten children increased their HTKS20 scores from fall (time 1) to spring (time 2).

The group × interaction in both models allowed us to determine whether there was a unique impact of schooling, over and above the effects of age. When comparing pre-k and kindergarten children, the group × time interaction was not significant, χ^2^(1) = 2.21, *p* = 0.14. While pre-k children who missed the cutoff appeared to show greater improvement in EF skills compared to kindergarten children who made the cutoff, this group × time interaction was not statistically significant. As shown in Figure 2, the slopes for pre-k and kindergarten children were not significantly different from each other. Table 4 Panel A presents the predicted outcomes, standard errors, and 95% confidence intervals for the model estimating kindergarten schooling effects on EF.

**TABLE 4:** Predicted outcomes for kindergarten and first grade schooling effects on HTKS scores.

**Cutoff status**	**Wave**	**Predicted outcome**	**SE**	**95% CI**
**Panel A: Kindergarten schooling effect (Predicted outcome: HTKS20)**				
Missed cutoff (pre-k)	Fall	14.01	1.24	11.59–16.45
Missed cutoff (pre-k)	Spring	16.90	0.66	15.60–18.19
Made cutoff (kindergarten)	Fall	17.54	0.43	16.69–18.39
Made cutoff (kindergarten)	Spring	18.42	0.37	17.69–19.14
**Panel B: First grade schooling effect (Predicted outcome: HTKS40)**				
Missed cutoff (kindergarten)	Fall	29.12	1.37	26.44–31.80
Missed cutoff (kindergarten)	Spring	34.06	0.99	32.12–36.01
Made cutoff (first grade)	Fall	34.62	0.63	33.39–35.86
Made cutoff (first grade)	Spring	36.51	0.40	35.72–37.29
**Panel C: Robustness check: Kindergarten schooling effect (Predicted outcome: HTKS40)**				
Missed cutoff (pre-k)	Fall	13.95	1.21	11.57–16.33
Missed cutoff (pre-k)	Spring	16.90	0.67	15.60–18.20
Made cutoff (kindergarten)	Fall	29.27	1.04	27.23–31.31
Made cutoff (kindergarten)	Spring	34.41	0.89	32.68–36.15

**FIGURE 3:**
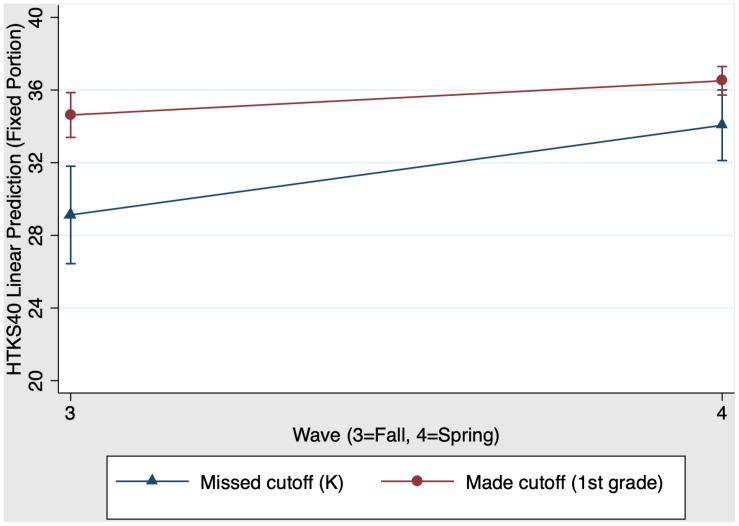
First grade schooling effects on fall-to-spring change in HTKS40 scores.

We also examined whether first grade schooling had a unique impact on children’s EF skills. Results showed that the overall model predicting HTKS40 scores was significant, χ^2^(3) = 26.75, *p* < 0.001. Figure 3 presents the first grade main effects and interaction on HTKS40 scores. There was a significant main effect of group, χ^2^(1) = 19.20, *p* < 0.001 (*f*^2^ = 0.11): First grade children who made the cutoff have stronger HTKS40 scores than their same-aged kindergarten peers who missed the cutoff for school entry the year before. There was also a significant main effect of time: χ^2^(1) = 11.97, *p* < 0.001 (*f*^2^ = 0.10): Both kindergarten and first grade children increased their HTKS40 scores from fall (time 3) to spring (time 4). In contrast to the previous model, the group × time interaction was significant when comparing kindergarten children and first grade children, χ^2^(1) = 4.20, *p* = 0.04 (*f*^2^ = 0.03). Specifically, kindergarten children who missed the cutoff for school entry the previous year show greater improvement in EF skills from fall to spring (time 3 to time 4) compared to first grade children who made the cutoff for school entry the previous year. As shown in Figure 3, the slope for kindergarten children was steeper than the slope for first grade children. Table 4 Panel B presents the predicted outcomes, standard errors, and 95% confidence intervals for the model estimating first grade schooling effects on EF.

### Are There Kindergarten and First Grade Effects on Reading?

**FIGURE 4:**
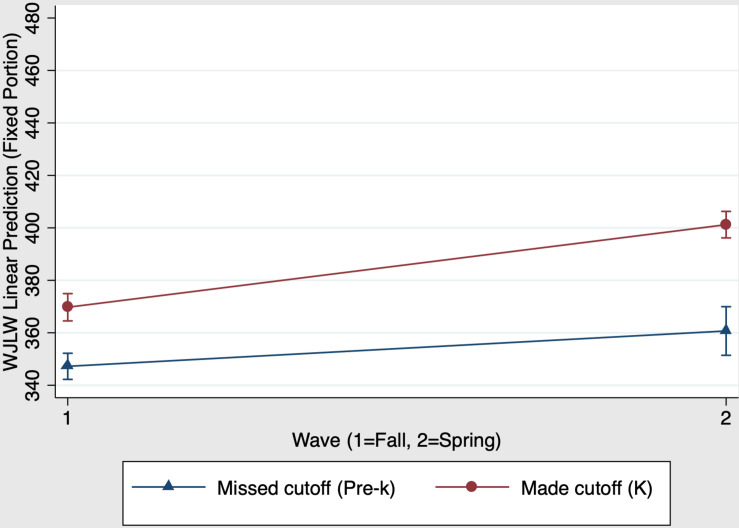
Kindergarten schooling effects on fall-to-spring change in Woodcock Johnson Letter-Word Identification W scores.

We examined whether kindergarten schooling had a unique impact on children’s reading skills. A mixed-effects regression with group, time, and group × time predicting children’s scores on the Woodcock Johnson Letter-Word Identification subtest was conducted. Results showed that the overall model predicting reading was significant, χ^2^(3) = 718.32, *p* < 0.001. Figure 4 presents graphs of the kindergarten main effects and interaction on reading scores. There was a significant main effect of group, χ^2^(1) = 50.75, *p* < 0.001 (*f*^2^ = 0.13): Kindergarten children who made the cutoff have stronger reading scores than their virtually same-aged pre-k peers who missed the cutoff. There was also a significant main effect of time, χ^2^(1) = 208.97, *p* < 0.001 (*f*^2^ = 0.38): Both pre-k and kindergarten children increased their reading scores from fall (time 1) to spring (time 2). When comparing pre-k and kindergarten children the group × time interaction was significant, χ^2^(1) = 36.53, *p* < 0.001 (*f*^2^ = 0.32). This is evidenced by the fact that kindergarten children show greater improvement from fall to spring compared to their same aged pre-k peers. Table 5 Panel A presents the predicted outcomes, standard errors, and 95% confidence intervals for the model estimating kindergarten schooling effects on reading.

**TABLE 5:** Predicted outcomes for kindergarten and first grade schooling effects on Woodcock Johnson Letter-Word Identification W scores.

**Cutoff status**	**Wave**	**Predicted outcome (WJLW W scores)**	**SE**	**95% CI**
**Panel A: Kindergarten schooling effect**				
Missed cutoff (pre-k)	Fall	347.22	2.54	342.24–352.20
Missed cutoff (pre-k)	Spring	360.69	4.73	351.41–369.96
Made cutoff (kindergarten)	Fall	369.73	2.65	364.53–374.93
Made cutoff (kindergarten)	Spring	401.23	2.58	396.18–406.27
**Panel B: First grade schooling effect**				
Missed cutoff (kindergarten)	Fall	373.13	9.46	354.59–391.68
Missed cutoff (kindergarten)	Spring	406.32	5.65	395.24–417.39
Made cutoff (first grade)	Fall	428.63	3.17	422.42–434.84
Made cutoff (first grade)	Spring	461.61	2.50	456.71–466.51

**FIGURE 5:**
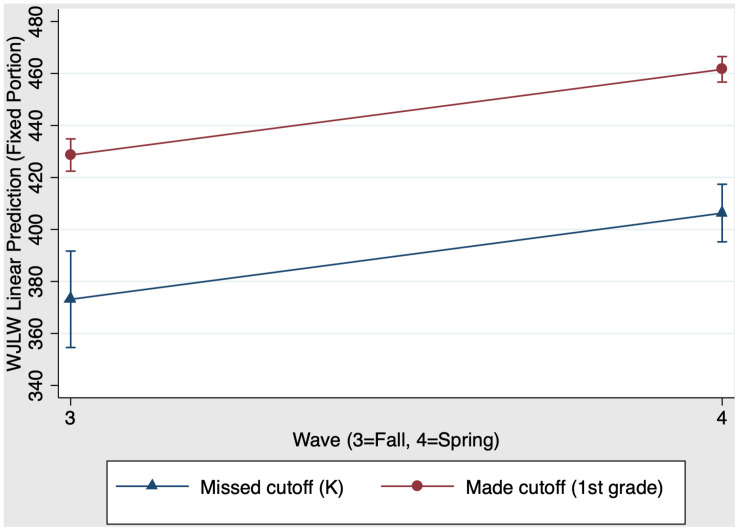
First grade schooling effects on fall-to-spring change in Woodcock Johnson Letter-Word Identification W scores.

We also examined whether first grade schooling had a unique impact on children’s reading skills. Results revealed that the overall model predicting reading was significant, χ^2^(3) = 476.75, *p* < 0.001. Figure 5 presents the first grade main effects and interaction on reading scores. There was a significant main effect of group, χ^2^(1) = 53.13, *p* < 0.001 (*f*^2^ = 0.12): First grade children who made the cutoff have stronger reading scores than their virtually same-aged kindergarten peers who missed the cutoff for school entry the year before. There was also a main effect of time, χ^2^(1) = 110.95, *p* < 0.001 (*f*^2^ = 1.01): Both kindergarten and first grade children increased their reading scores from fall (time 3) to spring (time 4). In contrast to the previous model, the group × time interaction was not significant when comparing kindergarten and first grade children, χ^2^(1) = 0.00, *p* = 0.97. In summary, we revealed that kindergarten schooling, but not first grade schooling, had a positive causal impact on reading growth from fall to spring. Table 5 Panel B presents the predicted outcomes, standard errors, and 95% confidence intervals for the model estimating first grade schooling effects on reading.

### Are There Kindergarten and First Grade Effects on Math?

**FIGURE 6:**
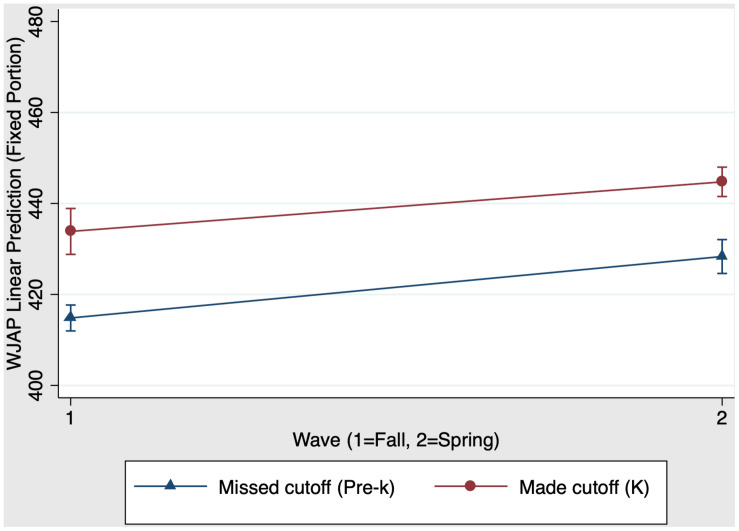
Kindergarten schooling effects on fall-to-spring change in Woodcock Johnson Applied Problems W scores.

Finally, we examined whether kindergarten schooling had a unique impact on children’s math skills. A mixed-effects regression with group, time, and group × time predicting children’s scores on the Woodcock Johnson Applied Problems subtest was conducted. Results showed that the model predicting math was significant, χ^2^(3) = 210.84, *p* < 0.001. Figure 6 presents the kindergarten main effects and interaction on math scores. There was a significant main effect of group, χ^2^(1) = 128.86, *p* < 0.001 (*f*^2^ = 0.21): Kindergarten children who made the cutoff have stronger math scores than their virtually same-aged pre-k peers who missed the cutoff. There was also a significant main effect of time, χ^2^(1) = 44.17, *p* < 0.001 (*f*^2^ = 0.50): Both pre-k and kindergarten children increased their math scores from fall (time 1) to spring (time 2). When comparing pre-k and kindergarten children, the group × time interaction was not significant, χ^2^(1) = 0.38, *p* = 0.54: Table 6 Panel A presents the predicted outcomes, standard errors, and 95% confidence intervals for the model estimating kindergarten schooling effects on math.

**TABLE 6:** Predicted outcomes for kindergarten and first grade schooling effects on Woodcock Johnson Applied Problems W scores.

**Cutoff status**	**Wave**	**Predicted outcome (WJAP W scores)**	**SE**	**95% CI**
**Panel A: Kindergarten schooling effect**				
Missed cutoff (pre-k)	Fall	414.83	1.45	411.99–417.68
Missed cutoff (pre-k)	Spring	428.34	1.90	424.62–432.06
Made cutoff (kindergarten)	Fall	433.85	2.58	428.81–438.90
Made cutoff (kindergarten)	Spring	444.76	1.65	441.53–447.98
**Panel B: First grade schooling effect**				
Missed cutoff (kindergarten)	Fall	433.01	2.75	427.62–438.41
Missed cutoff (kindergarten)	Spring	450.50	3.55	443.55–457.45
Made cutoff (first grade)	Fall	459.50	2.57	454.46–464.54
Made cutoff (first grade)	Spring	475.13	1.86	471.48–478.77

**FIGURE 7:**
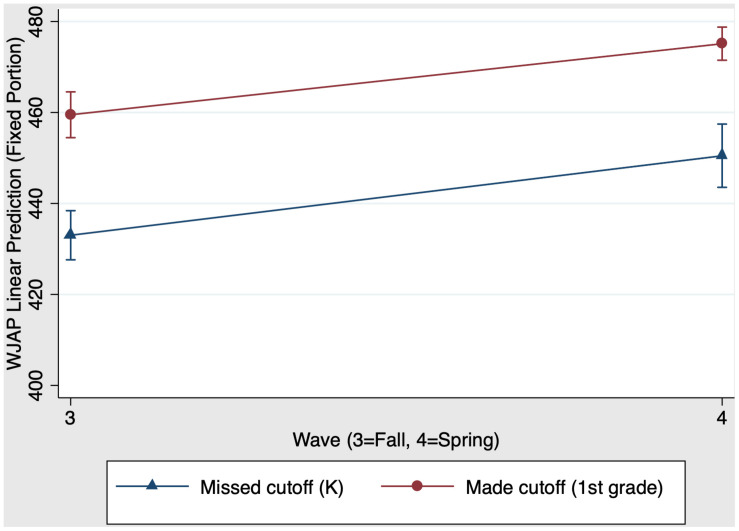
First grade schooling effects on fall-to-spring change in Woodcock Johnson Applied Problems W scores.

We also examined whether first grade schooling had a unique impact on children’s math skills. Results revealed that the overall model predicting math was significant, χ^2^(3) = 573.52, *p* < 0.001. Figure 7 presents the first grade main effects and interaction on math scores. There was a significant main effect of group, χ^2^(1) = 56.18, *p* < 0.001 (*f*^2^ = 0.23): First grade children who made the cutoff have stronger math scores than their virtually same-aged kindergarten peers who missed the cutoff for school entry the year before. There was also a significant main effect of time, χ^2^(1) = 153.04, *p* < 0.001 (*f*^2^ = 0.39): Both kindergarten children and first grade children increased their math scores from fall (time 3) to spring (time 4). When comparing kindergarten children and first grade children, the group × time interaction was not significant, χ^2^(1) = 0.26, *p* = 0.61. Table 6 Panel B presents the predicted outcomes, standard errors, and 95% confidence intervals for the model estimating first grade schooling effects on math. In summary, in contrast to the models predicting reading, the group × time interaction was not significant for both models predicting math.

### Robustness Checks

#### Using an Alternative Calculation of HTKS Scores

**FIGURE 8:**
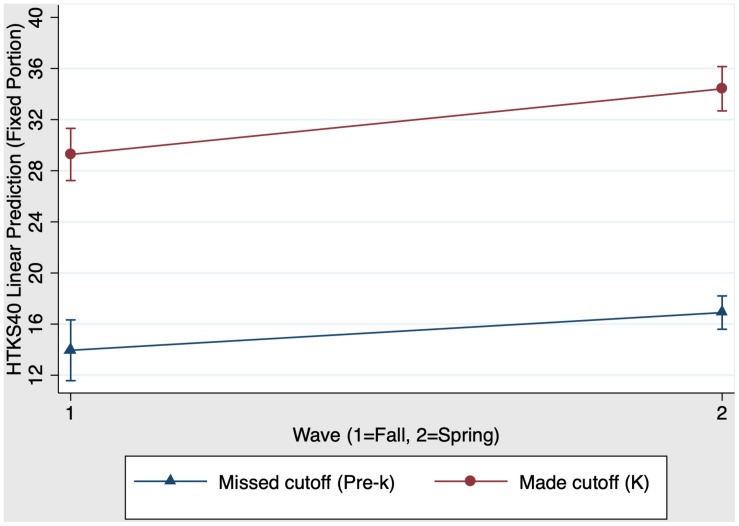
Robustness check: Kindergarten schooling effects on fall-to-spring change in HTKS40 scores.

We examined whether our kindergarten schooling results were sensitive to how children’s HTKS scores were calculated. As previously described, rather than considering only Part I of the HTKS for kindergarten children (i.e., HTKS20), an alternative method of measuring HTKS scores for kindergarten children was to consider their performance on both Parts I and II of the task, yielding a score out of 40 points (HTKS40). Results showed that this alternative model was significant, χ^2^(3) = 701.14, *p* < 0.001, indicating that the three-predictor model (group, time, and group × time) meaningfully predicted variability in HTKS40. Figure 8 shows the kindergarten main effects and interaction on HTKS40 scores. There was a significant main effect of group, χ^2^(1) = 237.59, *p* < 0.001 (*f*^2^ = 0.87): As before, kindergarten children who made the cutoff have stronger HTKS40 scores than their virtually same-aged pre-k peers who missed the cutoff. There was also a significant main effect of time, χ^2^(1) = 39.83, *p* < 0.001 (*f*^2^ = 0.08): Both pre-k and kindergarten children increased their HTKS40 scores from fall to spring. Finally, the group × time interaction was not significant, χ^2^(1) = 2.00, *p* = 0.16: The rate of change in EF between the two groups were not statistically different from each other. Table 4 Panel C presents the predicted outcomes, standard errors, and 95% confidence intervals for the model estimating kindergarten schooling effects on the HTKS40. In summary, our kindergarten schooling results did not depend on how the HTKS score was calculated for kindergarten children.

#### Sensitivity to Different Bandwidths

Our preferred bandwidth was a 2-month window before and after the cutoff. We also conducted a series of robustness checks by examining bandwidths of 1 and 3 months to examine whether our findings were sensitive to the size of the bandwidth. Supplementary Table S1 shows the contrasts of marginal linear predictions for the three bandwidths. Results were virtually unchanged—that is, effects that were not significant using a 2-month bandwidth remained not significant when the other bandwidths were used. Effects that were significant using a 2-month bandwidth remained significant for the other bandwidths, with the exception of the first grade schooling group × time interaction on HTKS scores, which did not reach our pre-specified significance threshold of *p* < 0.05 when 1- and 3-month bandwidths were used.

## Discussion

The present study used a school cutoff design to examine the unique effects of kindergarten and first grade schooling on children’s EF and academic skills. Quasi-experimental methods permit an examination of causal mechanisms that are not possible using correlational designs. A complex pattern of findings emerged. While children who made the cutoff for school entry performed better compared to children who missed the cutoff, growth in EF skills over the school year was strongest for kindergarten children who missed the cutoff for school entry the previous year compared to first grade children who made the cutoff the previous year. Turning to academic skills, kindergarten schooling, but not first grade schooling, had a unique causal impact on children’s growth in reading. However, no schooling effects on growth in math emerged.

### Schooling Effects on EF

Schooling-related influences uniquely predicted children’s EF skills as measured by the HTKS task. However, our findings were not entirely consistent with our predictions. We found that kindergarten children who missed the cutoff the previous year exhibited stronger EF growth compared to their same-aged first grade peers who made the cutoff the previous year. Therefore, while both groups improved their EF skills throughout the year, the rate of growth was strongest for the kindergarten children who missed the cutoff the year prior. In other words, there appears to be a “catch up effect” for these children.

We offer several interpretations to explain this finding. For some children, missing the cutoff for school entry means that they need to repeat an extra year of pre-k. This additional year of pre-k might be beneficial for some children who would benefit from a second year with the same teachers and caregivers. This continuity across years might allow children to continue to practice and build their behavioral regulation skills in a setting that is familiar and comfortable. In fact, a body of research has shown benefits for children attending 2 years of preschool compared to just one (e.g., Domitrovich et al., 2013). Therefore, this additional year of pre-k might catalyze greater growth in EF skills during kindergarten for these children. That said, we must acknowledge that we did not have access to information on previous childcare experiences in the present analysis. Subgroup analyses based on childcare histories would be an important contribution in future work.

The interpretation provided above does not downplay the importance of kindergarten in building behavioral skills. In fact, our data show that children who made the cutoff for school entry—both kindergarten children as well as first grade children who made the cutoff the previous year—experience stronger EF skills compared to their same-aged peers who missed the cutoff. Our data also show that both groups show growth in EF skills from fall to spring. While this indicates the potential role of biological maturation in the development of these skills, there could also be non-schooling-related factors, such as family/home influences also changing during this time, that might influence skill growth. Our data also appear to strengthen the argument that delayed school entry might not be detrimental to children’s skill growth, at least in the short term. Because this effect was not robust to different bandwidths, additional research to confirm and further characterize this finding is needed.

### Schooling Effects on Reading Skills

When examining pre-k and kindergarten children, we found that kindergarten schooling was a stronger predictor of children’s growth in decoding skills from fall to spring of the school year. This was evidenced by a steeper positive slope for kindergarten children who made the cutoff for school entry, compared to the pre-k children who missed the cutoff for school entry. Interestingly, when examining kindergarten and first grade children, the group × time interaction was not significant. That is, the slopes for kindergarten and first grade children were not significantly different from each other, indicating that both groups of children showed growth in reading skills at an equivalent rate. Our findings extend previous research using RDD that showed unique effects of pre-k, kindergarten, and first grade schooling on decoding skills (Kim and Morrison, 2018). That study examined schooling effects but was unable to examine growth in literacy skills across the school year due to the study’s particular implementation of the RDD, which examined differences in outcomes at a single point in time. The present study adds to this work by demonstrating that kindergarten children exhibit stronger growth in decoding skills during the year compared to their same-aged pre-k peers.

We offer one interpretation of this finding. It may be that there is a meaningful difference in reading instruction between pre-k and kindergarten, evidenced by the stronger growth in reading skills for kindergarten children compared to their same-aged pre-k peers. However, there appears to be no meaningful difference between kindergarten and first grade schooling as it relates to children’s growth in reading skills. Because kindergarten is often children’s first exposure to a formal schooling environment, the transition from pre-k to kindergarten might be more dramatic than the transition from kindergarten to first grade. This is consistent with research revealing that kindergarten is increasingly resembling first grade in terms of behavioral expectations and academic rigor (Bassok et al., 2016), suggesting that first grade might be simply a continuation of the academic emphasis that has begun in kindergarten (Indeed, this might partially explain why kindergarten children experience greater growth in EF skills compared to their first grade counterparts). In particular, the nature of reading instruction in first grade may not be markedly different from kindergarten. On the other hand, reading instruction in first grade may be focused on other aspects of literacy that is not captured by the test of decoding used in the present study. Future studies should incorporate classroom-level instructional data to elucidate the nature of the kindergarten schooling effect on children’s literacy skill development.

### Schooling Effects on Math Skills

Children’s scores on standardized tests of math achievement increased from fall to spring during kindergarten and first grade. However, we did not find unique influences of kindergarten or first grade on children’s math development. Our findings could point to the influence of non-schooling-related factors (e.g., age-related development, parenting, and the home environment) rather than specific schooling experiences. Another possibility for our null findings is the extremely short duration of math instruction; previous research has shown that less than 4 min per week are spent on math instruction in pre-k and kindergarten classrooms, compared to nearly an hour per week on literacy activities such as storybook reading (Skibbe et al., 2013). Because that study did not examine first grade children, we cannot determine whether or how first grade differs from earlier grades in terms of duration of instruction. However, if significantly more time is spent in math in first grade (or more time relative to reading), we might expect to see a unique first grade schooling effect, an effect we did not detect in the present study. This is a hypothesis that could be tested in future research.

Previous research has shown a positive effect of preschool intervention programs (Gormley et al., 2005; Weiland and Yoshikawa, 2013), as well as statewide preschool programs on children’s math development (Wong et al., 2008). While we did not examine preschool effects in the present study, it is interesting to consider why similar effects were not found in our study. There may be distinct benefits to implementing a specialized intervention program or a statewide curriculum; this was not the case in the present study. Another explanation is that while children’s math skills gradually develop across kindergarten and first grade, the impact of schooling on math development may become more prominent in later grades when greater emphasis is placed on explicit math instruction. Further, it is possible that kindergarten and first grade have an impact on rudimentary math skills such as cardinality and counting, and not on more advanced skills such as calculation and word problems. However, examining the impact on distinct aspects of math development was not possible using the Applied Problems subtest of the Woodcock Johnson. Future research would benefit from examining the impact of schooling using finer grained and developmentally sensitive measures of math skills.

### Limitations and Future Directions

While the school cutoff method permits causal inferences regarding the unique impact of schooling on children’s academic and behavioral outcomes, this approach is unable to discern what it is about schooling that causes improvements in these skills. Are schooling effects due to meaningful differences in instruction? Or are these impacts due to other factors such as classroom climate or peer effects? In addition to schooling effects, it would also be instructive to examine the potential role of children’s summer experiences in forming a fuller understanding of academic and behavioral skill growth. The present work adds to the small but growing literature regarding the causal impacts of regular schooling on children’s outcomes and lays the foundation for future studies that will ideally collect classroom-level data in order to elucidate the mechanisms of change that underlie the observed schooling effects.

We did not have access to teacher and classroom data that would help us understand the nature, type, quality, and duration of reading instruction in pre-k, kindergarten, and first grade classrooms. That is, what makes kindergarten reading instruction more effective than pre-k instruction, and why is first grade instruction not more effective than kindergarten instruction? Is the lack of early schooling effects on math achievement explained by the type of instructional activities, or the duration of instruction, or something else occurring during the first years of formal schooling? Without this information, we can only speculate as to why certain grade-level schooling experiences are more or less effective. Another limitation of the current study is our relatively small and convenient sample of participants. It is possible that subtle effects were not detected due to a lack of statistical power, especially with respect to the varying bandwidths used in the current study. Additionally, although children in the present study attended 13 elementary schools in the same school district, the sample was not representative of the larger population. The largely homogenous nature of the sample limits the generalizability of our results. Fortunately, large data sets such as the ECLS-K include a more diverse sample of children and contain information regarding school- and classroom-level data. Combined with information on children’s age at school entry, it is possible to examine whether and how differences in age at school entry, combined with instructional factors, impact different groups of young learners. This would be an important question for future research.

A strength of our study was that we examined pre-k, kindergarten, and first grade children across time. This allowed us to examine multiple grade-level schooling effects during the school transition period. However, greater attention must be placed on examining children beyond first grade, especially given our findings. For example, we found that kindergarten children experienced greater gains in EF compared to their same-aged first grade peers. We also found that there was no difference in reading growth rates between same-aged kindergarten and first grade children. What would these growth trajectories look like in the years that follow? That is, what are the longer-term cognitive and academic impacts of making (or missing) the cutoff date for school entry? And if there are any impacts, do those impacts depend on the nature of classroom instruction that children receive? These questions deserve greater consideration in order to better inform parents, educators, and policymakers regarding the impact of age-cutoff thresholds that school districts use to determine which children can and cannot enter school. Again, data sets that have repeated measures data on large groups of children across the elementary school years can provide ample opportunities to examine the longer-run impacts of variation in school entry.

## Data Availability Statement

The raw data supporting the conclusions of this article will be made available by the authors, without undue reservation, to any qualified researcher.

## Ethics Statement

The studies involving human participants were reviewed and approved by the University of Michigan IRB Health Sciences and Behavioral Sciences. Written informed consent to participate in this study was provided by the participants’ legal guardian/next of kin.

## Author Contributions

MK and SA led the conceptualization of the current study and drafted the manuscript. FM led the conceptualization of the original project from which data were collected. MK conducted data analysis. MK, SA, and FM contributed to interpreting the results, editing, and revising, and gave final approval of the version to be submitted. All authors contributed to the article and approved the submitted version.

## Conflict of Interest

The authors declare that the research was conducted in the absence of any commercial or financial relationships that could be construed as a potential conflict of interest.
