# Supplementary material for: The Effects of Kindergarten and First Grade Schooling on Executive Function and Academic Skill Development: Evidence From a School Cutoff Design
Source: Front Psychol. 2021 Jan 15;11:607973. doi: 10.3389/fpsyg.2020.607973 (PMC7874223; doi:10.3389/fpsyg.2020.607973)
Supplement: Supplementary file 1 [file Table_1.DOCX]

Supplementary Material

## Data structure

Due to the rolling nature of recruitment and data collection, there were separate cohorts of children examined in the present study. **Figure S1** shows how data were structured. In Year 2 of the study, there were two groups of children that were assessed—a group of children who missed the December 1 cutoff and were therefore in pre-k (Group A1), and another group of same-aged children who made the December 1 cutoff and enrolled in kindergarten (Group B1). These same children were followed into Year 3 of the study, when pre-k children entered kindergarten (Group C1—same set of children as Group A1) and kindergarten children entered first grade (Group D1—same set of children as Group B1). The same pattern occurred in Year 3—a new group of pre-k (Group A2) and kindergarten children (Group B2) were assessed in Year 3 and transitioned to kindergarten (Group C2) and first grade (Group D2), respectively, in Year 4.

As shown in the legend on the left-hand side, children in the A group (shaded in red) are the pre-k children who missed the cutoff, and children in the D group (shaded in blue) are the first grade children who made the cutoff. Importantly, there are two separate groups of kindergarten children—children in the B group are kindergarten children who made the cutoff in Years 2 and 3, and children in the C group are kindergarten children who missed the cutoff (i.e., these children were in pre-k the previous year).

As shown in the legend on the right-hand side, the schooling effects are determined by a comparison between two groups. Figure 1 shows that groups of the same letter are combined before the analysis is conducted. To illustrate, when calculating the kindergarten effect, children in Group A1 and A2 are combined and comprise the pre-k group; this group is compared against children in Groups B1 and B2 combined, which comprise the kindergarten group. In summary, the kindergarten effect is measured by comparing outcomes between children who *made* the cutoff for kindergarten entry (B group) compared to children who missed the cutoff and are in pre-k (A group). When calculating the first grade effect, the comparison is between first grade children who made the cutoff for kindergarten entry the previous year (D group) and kindergarten children who *missed* the cutoff the previous year (C group). Therefore, two separate groups of kindergarten children are used to calculate both schooling effects.


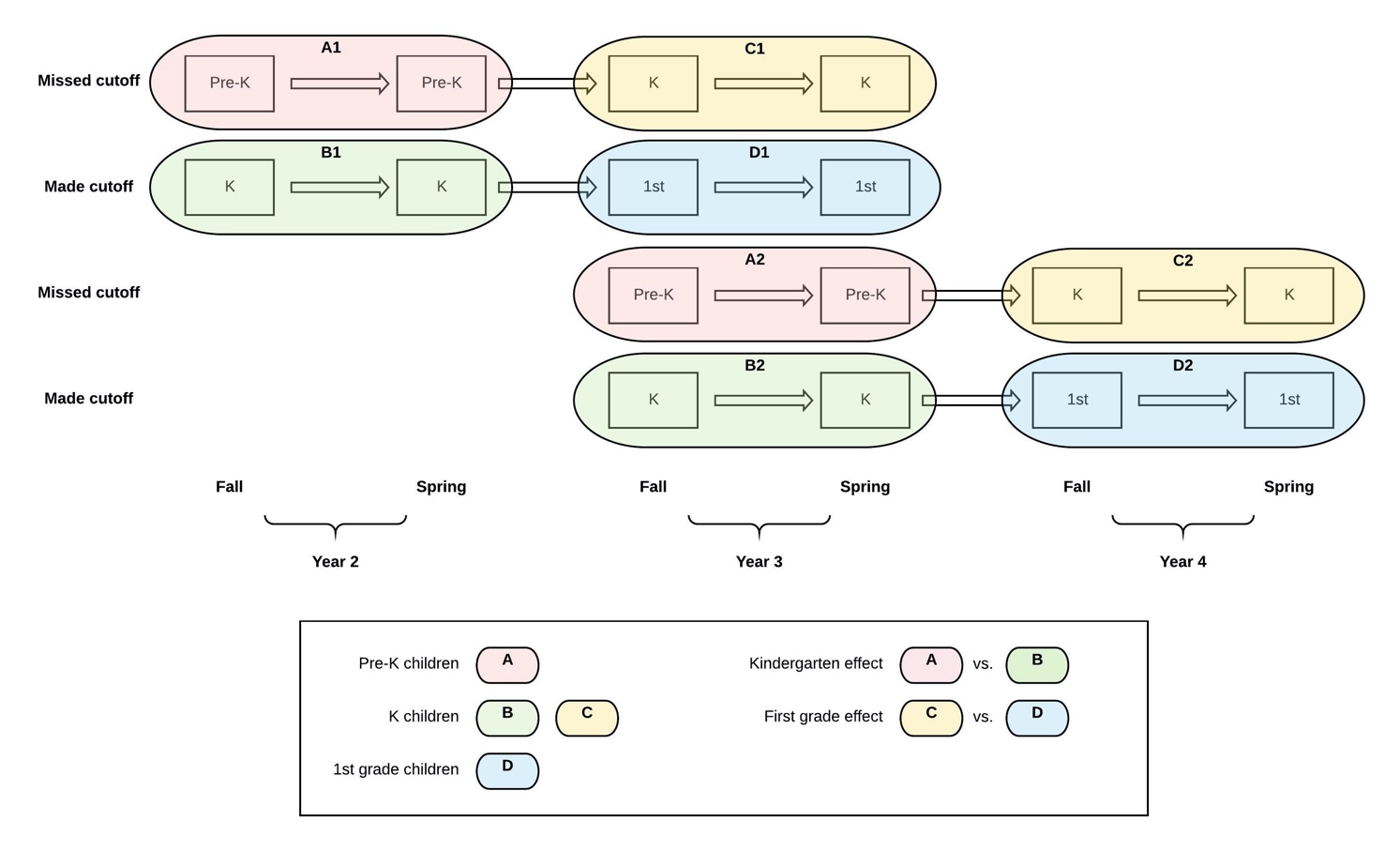


**Figure S1.** Longitudinal cohort data structure

**Table S1.** Robustness check: Contrasts of marginal linear predictions as a function of bandwidth

|  | **Group** | | | **Time** | | | **Group 🞨 Time** | | |
| --- | --- | --- | --- | --- | --- | --- | --- | --- | --- |
|  | **1mo** | **2mo** | **3mo** | **1mo** | **2mo** | **3mo** | **1mo** | **2mo** | **3mo** |
| Kindergarten effect on HTKS20 | **36.72** | **9.00** | **16.26** | **4.75** | **8.88** | **28.11** | 2.27 | 2.21 | 2.78 |
| First grade effect on HTKS40 | **3.89** | **19.20** | **65.99** | **3.81** | **11.97** | **13.20** | 0.02 | **4.20** | 3.30 |
| Kindergarten effect on reading | **57.41** | **50.75** | **47.49** | **539.90** | **208.97** | **176.28** | **32.85** | **36.53** | **21.22** |
| First grade effect on reading | **58.27** | **53.13** | **331.27** | **97.53** | **110.95** | **85.60** | 0.50 | 0.00 | 0.01 |
| Kindergarten effect on math | **86.33** | **128.86** | **283.29** | **29.42** | **44.17** | **41.81** | 0.50 | 0.38 | 0.29 |
| First grade effect on math | **45.45** | **56.18** | **132.42** | **88.64** | **153.04** | **80.57** | 0.83 | 0.26 | 0.01 |

Note. All coefficients represent chi-squared values with one degree of freedom [i.e., χ^2^(1)]. Bolded coefficients are significant at the .05 level.
